# Supplementary material for: A metabolic perspective of selection for fruit quality related to apple domestication and improvement
Source: Genome Biol. 2023 Apr 26;24:95. doi: 10.1186/s13059-023-02945-6 (PMC10131461; doi:10.1186/s13059-023-02945-6)
Supplement: Supplementary file 1 — Additional file 1: Fig. S1. Classificationand coefficient of variationvalues for all metabolites detected in apple flesh. Fig. S2. PCA analysis of all metabolite contents detected in other wild, M. sieversii, heirloom, and six apple cultivar groups. Fig. S3. Contents of total tannins and titratable acid in apple accessions among M. sieversii group, low-weight, and high-weight domesticated groups. Fig. S4. Co-mapped regions of soluble tannins identified by GWAS on Chr 16. Fig. S5. Differences between amino acid sequences of MdMyb9 and MD15G1051400. Fig. S6. Whole-genome screen for selective sweeps during apple domestication and improvement. The horizontal gray dotted lines indicate genome-wide thresholds that were estimated based on the top 5% of Fst values. Fig. S7. Co-mapped regions of LPE identified by GWAS on Chr 12. [file 13059_2023_2945_MOESM1_ESM.pdf]

Additional file 1: Fig. S1. Classification (a) and coefficient of variation (b) values for all metabolites detected in apple flesh. Coefficients of variations of each metabolite were independently calculated as follows: s/m, where ‘s’ and ‘m’ represent the standard deviation and mean of each metabolite in all accessions, respectively. Fig. S2. PCA analysis of all metabolite contents detected in other wild, *M. sieversii*, heirloom, and six apple cultivar groups. t[1] and t[2] represent the first and second principal component. R2x[1] and R2x[2] indicate the proportion of variance explained by t[1] and t[2], respectively. The colored dots indicate different apple accessions and the ellipses indicate the 95% confidence interval for each data set. Fig. S3. Fruit weight, total tannin content and titratable acid content in apple accessions among *M. sieversii* group, low-weight (≤ 90) domesticated group, and high-weight (≥ 300) domesticated group. ‘*’ indicates $p \leq 0.05$ by *t*-test. Fig. S4. Manhattan plots of procyanidin C1, procyanidin B4, arecatannin B1, cinnamtannin D1 and procyanidin A2 contents by GWAS. Co-mapped regions of the soluble tannins were identified on Chr 16. Fig. S5. Differences between amino acid sequences of *MdMyb9* and MD15G1051400 were marked by highlighting. Fig. S6. Whole-genome screen for selective sweeps during apple domestication and improvement. The horizontal gray dotted lines indicate genome-wide thresholds that were estimated based on the top 5% of Fst values. Fig. S7. Manhattan plots of LPE 18:1 and LPE 18:1 (2n isomer) contents by GWAS. Co-mapped regions of LPE contents were identified on Chr 12.

Additional file 1: Fig. S1

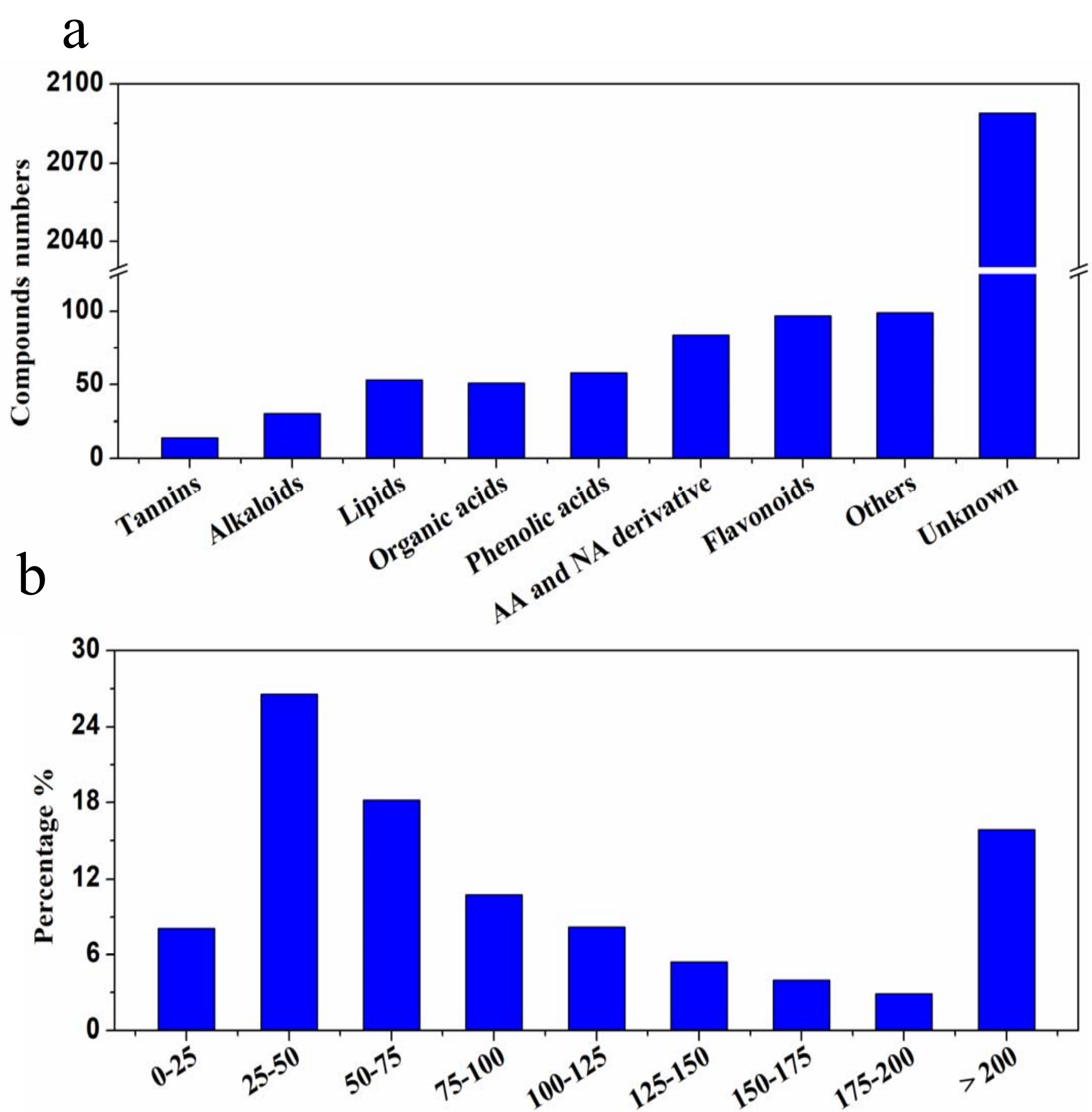

Additional file 1: Fig. S2

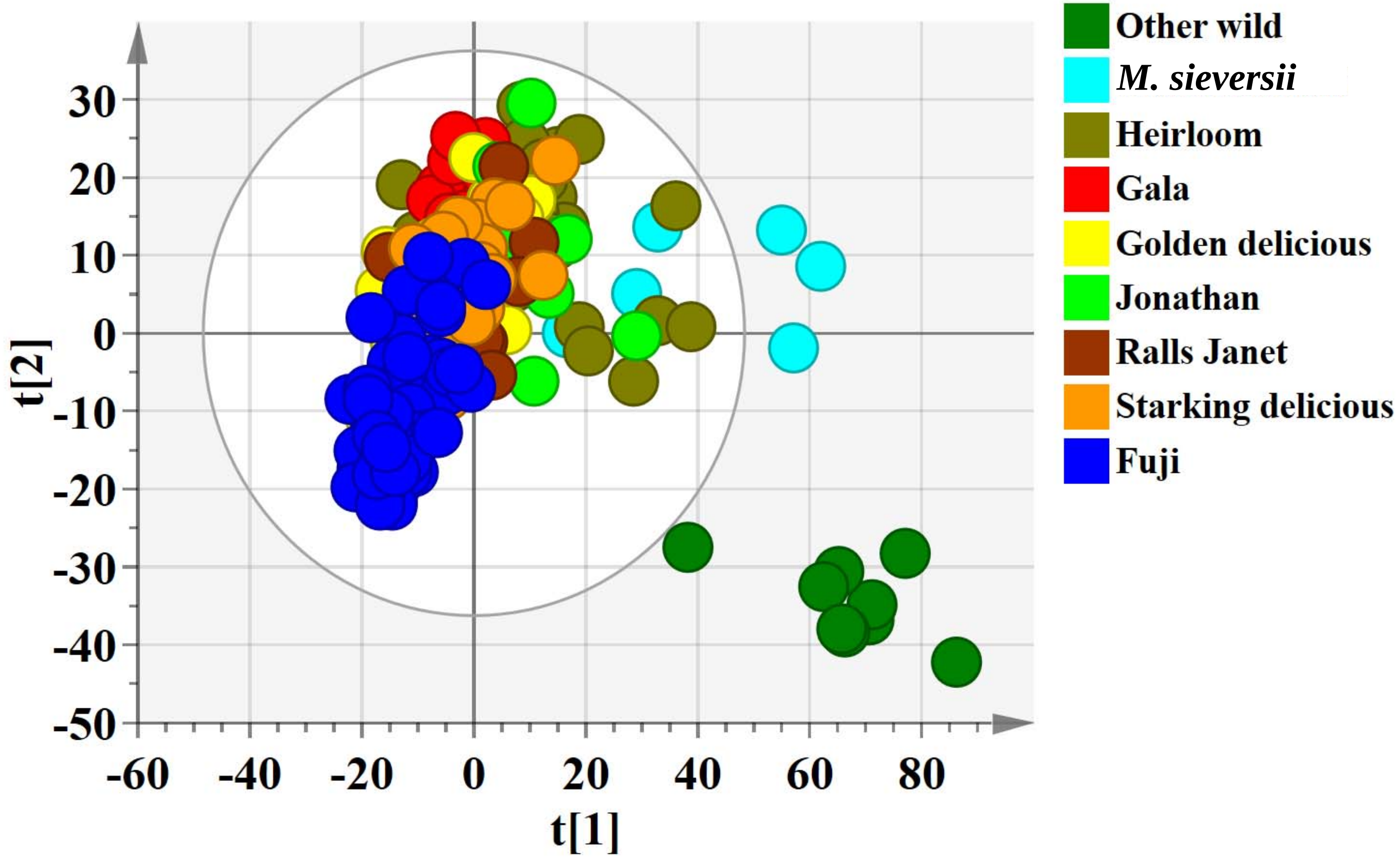

R2x[1] = 0.177 R2x[2] = 0.090

Ellipse: Hotelling's T2 (95%)

Additional file 1: Fig. S3

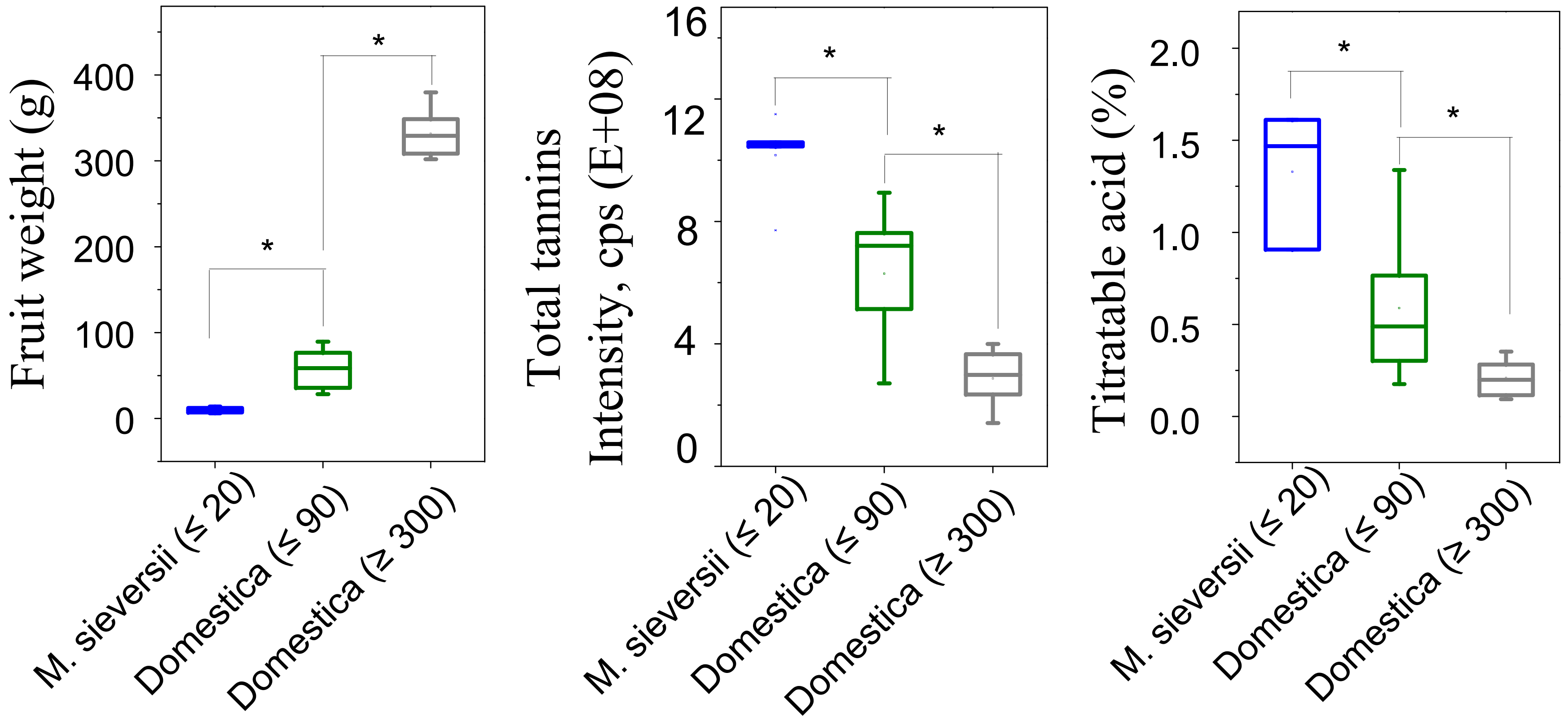

Additional file 1: Fig. S4

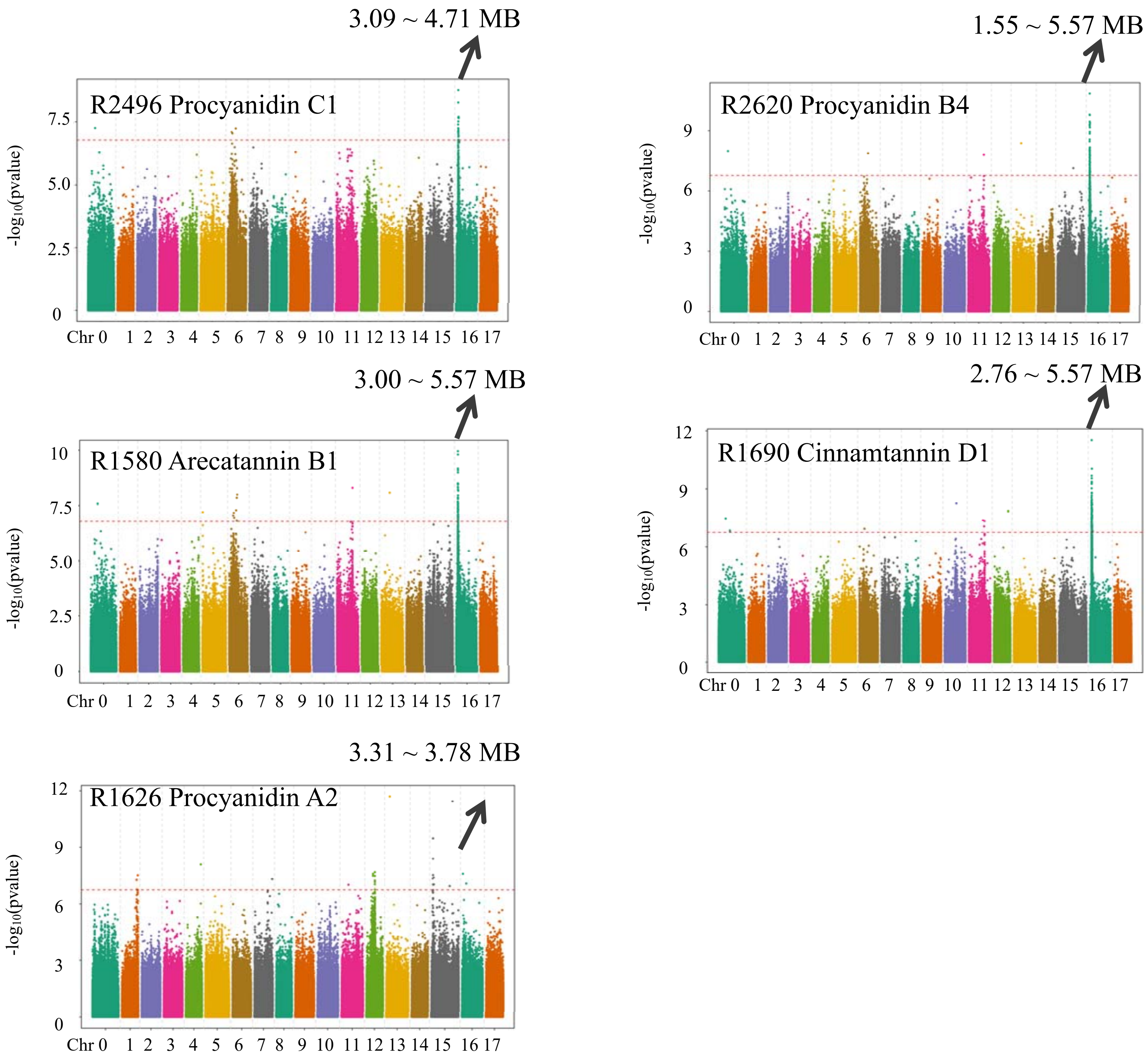

Additional file 1: Fig. S5

>MdMYB9

MGRSPCCSKEGLNRGAWTALEDKILSSYIKAHGEGKWRSLPKRAGLKRCGKSCRLRWLN
YLRPDIKRGNISGDEEELIVRLHNLLGNRWSLIAGRLPGRTDNEIKNYWNTTLGKKSKVDS
FSGSSKETSLNPCKSIAKKKDVESKT-STAAAQPLVIRTKATRLTKILVPQNIPSDENYTAAAA
NPLELQTQSAEKGGSTEEFPRTNAGDCSNILKNFGCDDDDIDAKGDQYCNEFQLLNSIPLDE
AMINDGCWTGGNGCDLEDYGASLDLDSLAFLLDSEEWPSQENVVV

>Myb9-like_MD15G1051400

MGRSPCCSKEGLNRGAWTALEDKILTAYIKAHGEGKWRSLPKRAGLKRCGKSCRLRWLN
YLRPDIKRGNISGDEEELIVRLHNLLGNRWSLIAGRLPGRTDNEIKNYWNTTLWKKSKADS
PSGSSKETSQHPSKSVVKKKDVESKTTSTAAAKPLVIRTKATRLSKILVPQNIPS-----------------
--------------------QGRS--

Additional file 1: Fig. S6

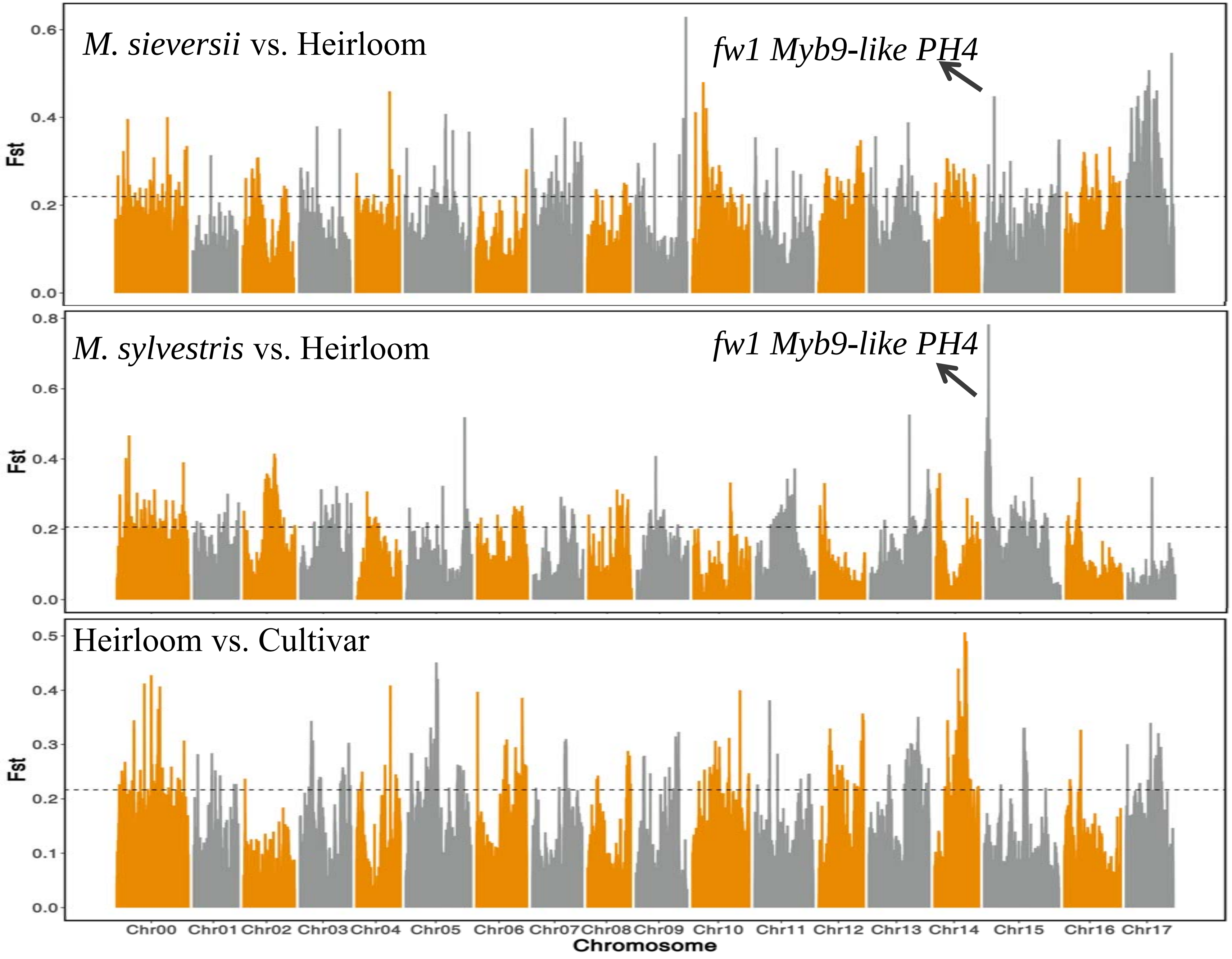

Additional file 1: Fig. S7

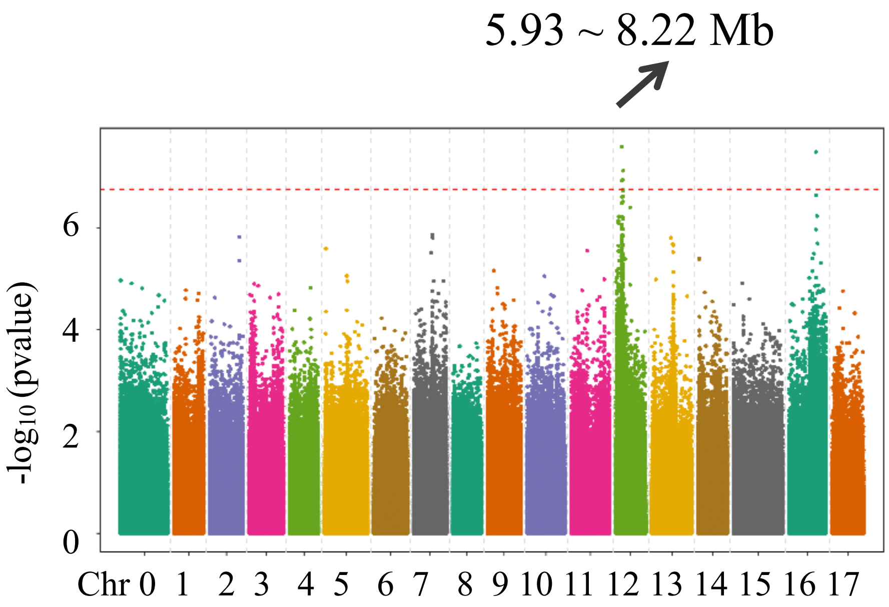

R2201 LPE 18:1

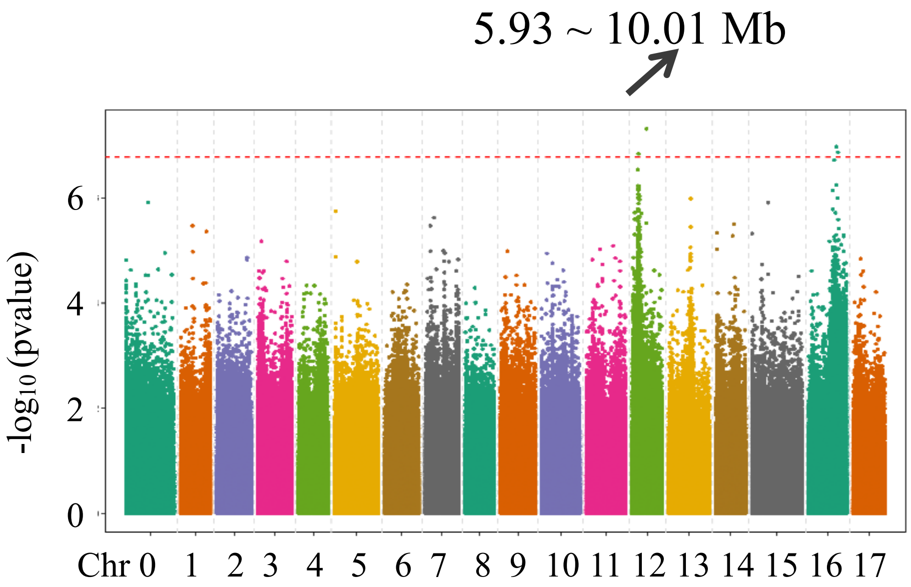

R1571 LPE 18:1 (2n isomer)
