# Supplementary material for: A metabolic perspective of selection for fruit quality related to apple domestication and improvement
Source: Genome Biol. 2023 Apr 26;24:95. doi: 10.1186/s13059-023-02945-6 (PMC10131461; doi:10.1186/s13059-023-02945-6)
Supplement: Supplementary file 3 — Additional file 3. Review history. [file 13059_2023_2945_MOESM3_ESM.docx]

Review History

**First round of review**

**Reviewer 1**

**Were you able to assess all statistics in the manuscript, including the appropriateness of statistical tests used?**

Yes, and I have assessed the statistics in my report.

**Comments to author:**

Manuscript of "A metabolic perspective of selection for fruit quality related to apple domestication and improvement" performed resequencing of 292 apple accessions, and selected parts of them for metabolism analysis. It found that the phylogenetic relationship and different metabolites in apple populations. Metabolites associated genetic loci were identified by mGWAS, and some genes related to traits of fruit quality were verified in tobacco leaves. This research will be meaningful for fruit study, but there are many problems like population selection and verified experiments need to be revised. It could be accepted for consideration of publication at present situation.

Major concerns,

Line 100-104, Line 130-132 : The authors should very carefully explain the criterions for sample selection in DNA sequencing ("292 apple accessions, including 10 M. sieversii, 7 M. sylvestris, 20 other wild species, 38 heirlooms, and 217 cultivars") and metabolism measurement ("269 accessions comprising 14 wild, 38 heirlooms, and 217 cultivars "). The wild and heirloom accessions counts 24/269 and 38/269, respectively. Will the sample selection have a bias for cultivars? Some test should be performed to obtain the similar result for phylogeny and metabolism, when no bias affect the number of accessions.

Fig. 1b, Line 110: Which is the best K value for this apple population? Could you explain the coincidence for the phylogenetic tree and the population structure? The authors should explain the significance for these apple accessions from K=2 to K=5.

Fig. 1d: What is the value of LD decay for different population? Does the LD decay of the five apple population have any correlation with nuclear diversity? Why?

Line 129: I wonder, if the authors use the metabolites to construct phylogenetic tree. Metabolite-based phylogeny would be different from the whole genome level, which will be very interesting to explore the differences.

Line 155-157: The different metabolites during domestication and improvement were identified, and the authors can find the selective sweep regions. The candidate mGWAS loci combine with selective sweeps should be analyzed.

Line 238-240: Why not do the overexpression of Myb9-like and LAR in apple callus or apple flesh? Somehow, tobacco leaves couldn't present the fruit flesh, and the stable and transient transformation system for apple is well-established.

Line 275-277: The same problem of transient overexpression in tobacco leaves instead of apple, which was used to verify the firmness of apple flesh.

Line 241: The SNP correlated with tennis content should be verified by experiment, such as Firefly Luciferase.

Line 321-325: Transient overexpression in tobacco leaves clarified the trait of fruit weight seems to be very weird. It should be clarified in apples.

Minor concerns,

Fig. 1a: Can't find the wild or heirlooms in the figure, but the contents in the main text are related. Please present the related other apple population in Fig. 1a.

Fig. 2a, d, e: Please carefully explain the figure legends for these figures, such as "t[1]", "t[2]", "R2x[1]", "R2x[2]", "Ellipse". What's the meaning of the circle? How about accessions inside or outside the circle? The front size for these figures differ from each other.

Fig. 3: Couldn't tell the P-value for some interesting significant loci.

Line 486: Do you use all the 2M SNPs for phylogenetic tree construction?

Line 493-496: Selective sweep calculation in Fig. 4d was presented by pi instead of Fst? Please double check the method.

**Reviewer 2**

**Were you able to assess all statistics in the manuscript, including the appropriateness of statistical tests used?**

No, I do not feel adequately qualified to assess the statistics.

**Comments to author:**

This is a high-quality piece of work that suffers from a rather narrow perspective. I feel that the authors have ignored a vast number of excellent genomics in other fruits including tomato which would have been excellent comparators to the apple and rendered the manuscript of general interest as is it is overly focussed on apples and likely has less broad appeal. I would suggest that the authors largely rewrite their discussion in the context of the many other studies on domestication of the metabolomes of crop species. Whilst this needs a major rewrite I have very few other comments. The English is excellent although there are a good few errors of syntax that should be carefully proofread for i.e. missing italics. In addition, I feel it important in such a study that the metabolomics data is reported following recent updates to reporting standards published last year in Nature Methods.

**Reviewer 3**

**Were you able to assess all statistics in the manuscript, including the appropriateness of statistical tests used?**

Yes, and I have assessed the statistics in my report.

**Comments to author:**

I found the study to be engaging and comprehensive. The methods were well described, particularly for the metabolite profiling. My main criticism of the work is the comparisons of domesticated apples with wild species, particularly those unrelated through domestication. I would recommend separating M. sieversii and M. sylvestris (primary progenitors) from other wild apples. Otherwise we cannot boldly state the metabolite variation is due to selection through domestication.

I've made comments pointing at a few specific lines in your MS, but the whole should be checked where these claims are made.

My comments above apply here as well. I also have some hesitation about the strength of some of the claims for LPE content being "crucial" for fruit storage. I think the methods show some promise for the role of LPE but more elaborate studies need to be conducted.

I think the methods are well described. I would also encourage including accession level information from the repository, so individuals could request the exact germplasm for follow up studies.

Other metabolites studies have been conducted, but few cover the breadth of chemical and genetic approaches.

Though focused primarily on apple, the approaches used could be useful for other perennial fruit crops.

**Authors Response**

**Point-by-point responses to the reviewers’ comments:**

Reviewer #1:

Manuscript of "A metabolic perspective of selection for fruit quality related to apple domestication and improvement" performed resequencing of 292 apple accessions, and selected parts of them for metabolism analysis. It found that the phylogenetic relationship and different metabolites in apple populations. Metabolites associated genetic loci were identified by mGWAS, and some genes related to traits of fruit quality were verified in tobacco leaves. This research will be meaningful for fruit study, but there are many problems like population selection and verified experiments need to be revised. It could be accepted for consideration of publication at present situation.

Major concerns,

Line 100-104, Line 130-132 : The authors should very carefully explain the criterions for sample selection in DNA sequencing ("292 apple accessions, including 10 M. sieversii, 7 M. sylvestris, 20 other wild species, 38 heirlooms, and 217 cultivars") and metabolism measurement ("269 accessions comprising 14 wild, 38 heirlooms, and 217 cultivars "). The wild and heirloom accessions counts 24/269 and 38/269, respectively. Will the sample selection have a bias for cultivars? Some test should be performed to obtain the similar result for phylogeny and metabolism, when no bias affect the number of accessions.

Fig. 1b, Line 110: Which is the best K value for this apple population? Could you explain the coincidence for the phylogenetic tree and the population structure? The authors should explain the significance for these apple accessions from K=2 to K=5.

Fig. 1d: What is the value of LD decay for different population? Does the LD decay of the five apple population have any correlation with nuclear diversity? Why?

Line 129: I wonder, if the authors use the metabolites to construct phylogenetic tree. Metabolite-based phylogeny would be different from the whole genome level, which will be very interesting to explore the differences.

Line 155-157: The different metabolites during domestication and improvement were identified, and the authors can find the selective sweep regions. The candidate mGWAS loci combine with selective sweeps should be analyzed.

Line 238-240: Why not do the overexpression of Myb9-like and LAR in apple callus or apple flesh? Somehow, tobacco leaves couldn't present the fruit flesh, and the stable and transient transformation system for apple is well-established.

Line 275-277: The same problem of transient overexpression in tobacco leaves instead of apple, which was used to verify the firmness of apple flesh.

Line 241: The SNP correlated with tennis content should be verified by experiment, such as Firefly Luciferase.

Line 321-325: Transient overexpression in tobacco leaves clarified the trait of fruit weight seems to be very weird. It should be clarified in apples.

Minor concerns,

Fig. 1a: Can't find the wild or heirlooms in the figure, but the contents in the main text are related. Please present the related other apple population in Fig. 1a.

Fig. 2a, d, e: Please carefully explain the figure legends for these figures, such as "t[1]", "t[2]", "R2x[1]", "R2x[2]", "Ellipse". What's the meaning of the circle? How about accessions inside or outside the circle? The front size for these figures differ from each other.

Fig. 3: Couldn't tell the P-value for some interesting significant loci.

Line 486: Do you use all the 2M SNPs for phylogenetic tree construction?

Line 493-496: Selective sweep calculation in Fig. 4d was presented by pi instead of Fst? Please double check the method.

*For the population selection, we considered as many wild species and cultivar types as possible to cover the apple domestication and improvement process. In the manuscript, the apple accession included five major groups, namely other wild accessions, M. sieversii, M. sylvestris, heirlooms, and cultivars. The cultivars were further grouped into 6 clades, namely ‘Jonathan’, ‘Gala’, ‘Golden Delicious’, ‘Starking Delicious’, ‘Ralls Janet’, and ‘Fuji’ pedigrees, respectively. These cultivars represent different pedigrees and consumption types. As described in previous reports, M. sieversii is the most important progenitor of apple domestication, followed by intensive introgressions from M. sylvestris (Duan et al., 2017; Liao et al., 2021). We compared the whole accessions of other wild, M. sieversii, M. sylvestris, heirloom, and cultivars at the genomic level and obtained similar results to previous reports (Duan et al., 2017; Liao et al., 2021). The M. sylvestris is a European wild species that has not been deposited in the National Repository of Apple and Pear Germplasm Resources in China. Thus, at the metabolic level, we compared the other wild accessions, M. sieversii, heirlooms, and cultivars. According to the report from Liao et al., the comparisons between M. sieversii and heirloom, and between heirloom and cultivar represent the apple domestication and improvement process, respectively. In addition, the DNA was obtained from the young leaves of each accession using a Plant DNA Mini Kit (Aidlab Biotech Ltd., China). After constructing the DNA library, an Illumina HiSeq XTen/NovaSeq/BGI platform was used for sequencing, with 150 bp read lengths, conducted by a credible service provider (Biomarker Technologies, China). For the metabolite measurement, apple flesh was freeze-dried and ground for 1 min at 30 Hz using a mixer mill (MM 400, Retsch, Germany) with zirconia beads. A total of 80 mg of flesh powder was used for further analysis. The above information has been added to the manuscript or the additional files (Lines 462-466, 485-497, 512-517).*

*Thanks for the comments. In the revised manuscript, the transient over-expressions of LAR, Myb9-like, FAD2, NAP, and ABCG25 in apple fruits were conducted. From the results, transiently overexpressing Myb9-like and LAR exhibited a significantly higher level of procyanidins in apple fruits than the controls (Figs. 4f and 4g). Transient overexpression of FAD2 in apple fruit led to a decreased level of LPE 18:1 than that of the control (Fig. 5g). Transiently overexpressing NAP in apple fruits indicated that SA content was significantly increased, which further supports the role of NAP in SA synthesis (Fig. 6l). Also, apple fruits transiently overexpressing ABCG25 contained a significantly higher ABA content than the controls (Fig. 6m). The related results were added to the manuscript (Lines 246-248, 286-287, 328-329, 331-332).*

Reviewer #2:

Comment: This is a high-quality piece of work that suffers from a rather narrow perspective. I feel that the authors have ignored a vast number of excellent genomics in other fruits including tomato which would have been excellent comparators to the apple and rendered the manuscript of general interest as it is overly focused on apples and likely has less broad appeal. I would suggest that the authors largely rewrite their discussion in the context of the many other studies on domestication of the metabolomes of crop species. Whilst this needs a major rewrite I have very few other comments. The English is excellent although there are a good few errors of syntax that should be carefully proofread for i.e. missing italics.

*Reply: Thanks for the comments. The discussion part has been revised to compare other studies on the domestication of the metabolomes of crop species (Lines 342-348, 363-374, 375-391, 432-444). Other parts have also been revised to improve the quality of the manuscript. The revised parts have been highlighted in the manuscript.*

Comment: In addition, I feel it important in such a study that the metabolomics data is reported following recent updates to reporting standards published last year in Nature Methods.

*Reply: The metabolomics data has been provided in Additional file 2: Table S4 according to the reporting standards in Nature Methods (Alseekh, S., et al. Mass spectrometry-based metabolomics: a guide for annotation, quantification and best reporting practices, 2021. DOI: 10.1038/s41592-021-01197-1).*

Reviewer #3:

Comment: I found the study to be engaging and comprehensive. The methods were well described, particularly for the metabolite profiling. My main criticism of the work is the comparisons of domesticated apples with wild species, particularly those unrelated through domestication. I would recommend separating M. sieversii and M. sylvestris (primary progenitors) from other wild apples. Otherwise we cannot boldly state the metabolite variation is due to selection through domestication.

*Reply: We have re-analyzed the data according to the comments. The apple accession has been divided into five major groups, including other wild, M. sieversii, M. sylvestris, heirloom, and cultivar groups. As described in previous reports, M. sieversii is the most important progenitor of apple domestication, followed by intensive introgressions from M. sylvestris (Duan et al., 2017; Liao et al., 2021). We compared the whole accessions of other wild, M. sieversii, M. sylvestris, heirloom, and cultivars at the genomic level and obtained similar results to previous reports (Duan et al., 2017; Liao et al., 2021). The M. sylvestris is a European wild species that has not been deposited in the National Repository of Apple and Pear Germplasm Resources in China. Thus, at the metabolic level, we compared the other wild accessions, M. sieversii, heirlooms, and cultivars. According to the report from Liao et al., the comparisons between M. sieversii and heirloom, and between heirloom and cultivar represent the apple domestication and improvement process, respectively. The manuscript has been revised according to the re-analyzed results (Lines 132-133, 144-145, 152-156, 159-160, 162-167, 212-213, 226-230, 233, 385-387, 537-538).*

Comment: I've made comments pointing at a few specific lines in your MS, but the whole should be checked where these claims are made.

*Reply: The comments (1-15) in MS were listed and replied to point-by-point below. Also, other related problems were revised and marked by highlighting in the manuscript.*

Comment: 1. Line 130: Why only flesh? Typically the peel is also done?

*Reply: We also discussed this question extensively before we collected the samples. The ratio of flesh and peel is different among apple accessions, especially in the very big and very small fruits. The metabolite content in the flesh and peel of an apple is extremely different according to our previous report (Liang, X., et al. 2020, DOI: 10.3390/molecules25184153). In flesh, the soluble metabolites are more abundant while the un-soluble metabolites like polysaccharides are of high concentration in the peel. Therefore, the peeled fruit samples are much more uniform than the no-peeled fruit. In addition, we mainly focused on the edible quality in this study, which is largely affected by soluble metabolites rather than the un-soluble metabolites. Also, customers in China often peel the fruit before they eat an apple. Thus, the flesh was selected for metabolites analysis in this work. In the future, we will analyze the processing quality of apples among cultivars, which is mainly affected by the metabolites in the peel.*

Comment: 2. Line 145: Can we really say these cultivars represent the historic selection and domestication of apple. These are more the focus of breeding the last several decades.

*Reply: The sentence has been revised as ‘The results obtained from metabolite differentiation were generally consistent with the phylogenetic tree relationships, indicating a strong relationship between changes in metabolite and genomic variation of apples’ (Lines 147-149).*

Comment: 3. Line 189: Difficult to standardize tissues between domesticated apples and wild apples. Could it be due to additional juiciness of the fruit (dilution effect) than differences in metabolite production? I think the claims will be strengthened if you compare the domesticated to M. sieversii which is most similar in fruit quality.

*Reply: In this research, we used the same amount of freeze-dried apple powders for metabolites analysis, the effect of juiciness dilution has been largely avoided. The domesticated apples with high- and low-weight were compared, as well as the wild and domesticated fruit were compared. Both comparisons showed a decreasing trend of tannins and organic acids. Additionally, in the revised manuscript, we compared M. sieversii and domesticated apples, and the results showed that the tannins and organic acids are significantly higher in M. sieversii (Lines 162-164).*

Comment: 4. Line 190 ‘suggesting that tannin and organic acid content were inversely correlated with the selection of enhanced fruit weight.’. Need to more carefully separate human selection (domestication) and evolutions. It's clear that Malus species evolved divergent fruit types for seed dispersal. These metabolite variations are more likely due to species variation than human selection.

*Reply: The sentence has been changed to ‘were inversely correlated with the evolution of enhanced fruit weight.’ (Line 195).*

Comment: 5. Line 202: Indicate units for chromosome axes.

*Reply: The unit for chromosome length is Mb, which has been added in the figure legend (Line 210).*

Comment: 6. Line 208 ‘Tannins, including procyanidins (A1, A2, B4, and C1) and gallic acid O-hexoside, were accumulated to higher levels in the wild group than heirlooms and cultivars’. Again, so much diversity in the wild unrelated to apple domestication. I would suggest repeating the analysis separating wild progenitors from other wild Malus.

*Reply: We have compared the primary progenitor M. sieversii and heirloom, and found that the procyanidins (A1, A2, B4, B5, and C1) were accumulated to higher levels in the M. sieversii group. The sentence has been revised as ‘Tannins, including procyanidin A1, procyanidin A2, procyanidin B4, procyanidin B5, and procyanidin C1, were accumulated to higher levels in the M. sieversii group than in heirlooms and cultivars’ (Lines 212-214).*

Comment: 7. Line 212: Khan SA, Schaart JG, Beekwilder J, Allan AC, Tikunov YM, Jacobsen E, et al. (2012) The mQTL hotspot on linkage group 16 for phenolic compounds in apple fruits is probably the result of a leucoanthocyanidin reductase gene at that locus. BMC Research Notes 5:618. https://doi.org/10.1186/1756-0500-5-618

*Reply: The reference has been added to the manuscript (Line 218, 718-720).*

Comment: 8. Line 303: Identify apples in figure.

*Reply: ‘High weight’ and ‘Low weight’ were added in Fig. 6a (Line 311).*

Comment: 9. Line 348 ‘In this study, apple domestication resulted in the reduction of certain compounds that produce adverse effects on taste’. See earlier comment about evolution vs domestication.

*Reply: We re-analyzed the distinct metabolites between the primary progenitor M. sieversii and heirloom, and a total of 595 different metabolites were identified (Additional file 2: Table S5), revealing the metabolome variation in apples during domestication (Lines 159-162).*

Comment: 10. Line 371 ‘We demonstrated that lysolipid LPE 18:1 is crucial for maintaining the firmness of apple’. Not comfortable with the word choice here. The results are compelling, but I don't think provide definitive evidence that LPE is Crucial. There are other molecular tests (transient expression in apples e.g.), especially in additional cultivars to show the trend across apple cultivars.

*Reply: The FAD2 transient overexpression in apples has been analyzed in the revised manuscript. The results showed that transient overexpression of FAD2 in apple fruit led to a decreased level of LPE 18:1 than that of the control (Fig. 5g). Also, a close correlation between the content of LPE 18:1 and the firmness of cultivated apple accessions was investigated (Fig. 5e). In the manuscript, ‘is crucial for’ has been revised as ‘is potentially maintaining’ (Lines 53, 297, 399).*

Comment: 11. Line 382: This is comparing SA and ABA across species, and other biological factors may be related to fruit size. Could you do comparisons among the domesticated fruit?

*Reply: The comparison of high-weight and low-weight was analyzed among domesticated apples. We have revised the sentence as ‘The potential metabolites that could affect fruit weight were investigated among domesticated apples.’ (Lines 158, 186-187, 313, 386, 412).*

Comment: 12. Line 417: is the accession information included in Supplementary Tables? I only saw lab IDs. Adding this will allow others to follow up.

*Reply: Thanks for the kind suggestion. The accession name supplied is unique in the National Repository of Apple and Pear Germplasm Resources. The accession information is not available for now. If others would like to follow up on the research, they can contact by email: wangdajiang@caas.cn.*

Comment: 13. Line 421 ‘3 M. baccata, 3 M. sieboldii, 7 M. spectabilis, and 6 M. toringoides’. These are interesting to include but are very divergent from M. domestica and wild progenitors.

*Reply: The divergent wild species were named ‘other wild’, and the differences in genomic level were comprehensively compared among accessions of other wild, M. sieversii, M. sylvestris, heirloom, and cultivars to draw a complete phylogenetic tree. However, for the metabolite profiling, not all the wild species can be obtained, particularly the M. sylvestris that is not deposited in China. We compared the metabolic difference among other wild accessions, M. sieversii, heirlooms, and cultivars. The results showed that other wild accessions were divergent from other groups, thus, we compared the metabolite differentiation of M. sieversii, heirlooms, and cultivars, and also compared the metabolic difference among different apple pedigrees.*

Comment: 14. Line 422: I think freeze drying the samples would have given better comparisons between species.

*Reply: The samples were immediately frozen in liquid nitrogen and stored at -80 °C. After all the fruit samples were prepared, the samples were freeze-dried and used for further measurement (Line 485-486).*

Comment: 15. Line 440: This part is well described.

*Reply: Thanks for the comments.*

Comment: Other metabolites studies have been conducted, but few cover the breadth of chemical and genetic approaches.

*Reply: Thanks for the comments.*

Comment: Though focused primarily on apple, the approaches used could be useful for other perennial fruit crops.

*Reply: Thanks for the comments.*

**Second round of review**

**Reviewer 1**

The authors added valuable experimental data for apple transient over-expression. The authors has well addressed most of my concerns. The manuscript is suitable accepted for publication.

I am adding only a few minor suggestions for improvement:

Fig. 1a: Can't find the wild or heirlooms in the figure, but the contents in the main text are related. Please present the related other apple population in Fig. 1a.

Fig. 2a, d, e: Please carefully explain the figure legends for these figures, such as "t[1]", "t[2]", "R2x[1]", "R2x[2]", "Ellipse". What's the meaning of the circle? How about accessions inside or outside the circle? The front size for these figures differ from each other.

Fig. 3: Couldn't tell the P-value for some interesting significant loci.

**Reviewer 2**

I am satisified by the changes made by the authors. Two very minor comments about their additions (i) numbers below 10 should be spelt out in letters e.g. 9 should rather be nine etc (ii) the O´s in many secondary metabolite names need to be italicized.

**Reviewer 3**

Thank you for the opportunity to review. I found this version to be substantially improved from the original version. All my criticisms were addressed by the authors, and after reviewing the comments of the other referees, I believe they have sufficiently addressed those as well. This will be a meaningful contribution to understanding the domestication history of apple.

**Authors Response**

**Point-by-point responses to the reviewers’ comments:**

Reviewer #1: The authors added valuable experimental data for apple transient over-expression. The authors has well addressed most of my concerns. The manuscript is suitable accepted for publication.

I am adding only a few minor suggestions for improvement:

Fig. 1a: Can't find the wild or heirlooms in the figure, but the contents in the main text are related. Please present the related other apple population in Fig. 1a.

*Reply: Geographic distribution of other wild, M. sylvestris, M. sieversii, heirloom, and cultivar has been added in Fig 1a. The circle represents the sample size.*

Fig. 2a, d, e: Please carefully explain the figure legends for these figures, such as "t[1]", "t[2]", "R2x[1]", "R2x[2]", "Ellipse". What's the meaning of the circle? How about accessions inside or outside the circle? The front size for these figures differ from each other.

*Reply: t[1] and t[2] represent the first and second principal component. R2x[1] and R2x[2] indicate the proportion of variance explained by t[1] and t[2], respectively. The colored dots indicate different apple accessions and the ellipses indicate the 95% confidence interval for each data set. The circle indicates the confidence interval. The above information has been added in the figure legend of Fig. 2. The front in Fig. 2a, 2d, 2e has been revised to the same size.*

Fig. 3: Couldn't tell the P-value for some interesting significant loci.

*Reply: The P-value of all significant SNPs associated with apple metabolites were listed in Additional file 2: Table S9. This information has been added in the legend of Fig. 3 in the manuscript.*

Reviewer #2: I am satisified by the changes made by the authors. Two very minor comments about their additions (i) numbers below 10 should be spelt out in letters e.g. 9 should rather be nine etc. (ii) the O´s in many secondary metabolite names need to be italicized.

*Reply: Thanks for the comments. The numbers below 10 were spelt out all through the manuscript. O´s in many secondary metabolite names were italicized in the manuscript and additional files. Other grammar errors were also revised.*

Reviewer #3: Thank you for the opportunity to review. I found this version to be substantially improved from the original version. All my criticisms were addressed by the authors, and after reviewing the comments of the other referees, I believe they have sufficiently addressed those as well. This will be a meaningful contribution to understanding the domestication history of apple.

*Reply: Thanks for your efforts in reviewing this manuscript.*
